# Research advances on acute kidney injury and brain dysfunction

**DOI:** 10.3389/fneph.2026.1867473

**Published:** 2026-07-23

**Authors:** Min Li, Xiaohui Liao, Jingyang Ran

**Affiliations:** Department of Nephrology, The Second Affiliated Hospital of Chongqing Medical University, Chongqing, China

**Keywords:** acute kidney injury, AKI-associated brain dysfunction, kidney–brain axis, uremic toxins, neuroinflammation, insulin resistance, multimodal neuroimaging, machine learning

## Abstract

Acute kidney injury (AKI) is a prevalent critical clinical condition primarily characterized by a sharp deterioration of renal function within 48 hours. Accumulating clinical evidence has demonstrated that AKI predisposes patients to multisystem complications, among which renal–brain axis dysfunction is well recognized. This condition manifests as delirium, coma and stroke, also elevates the risk of chronic cognitive impairment, dementia and depression. Such complications not only increase short-term all-cause mortality but also severely impair the long-term quality of life in survivors, thereby placing a substantial socioeconomic burden on global healthcare systems. Current mechanistic investigations into AKI-associated brain dysfunction have predominantly centered on canonical pathological pathways, including systemic inflammatory response, oxidative stress, hippocampal neuronal injury, uremic toxin accumulation and others. Emerging evidence indicates that insulin resistance (IR) is highly prevalent in AKI patients and closely correlated with the pathophysiological progression of AKI. We will discuss the specific role of IR in AKI-induced cognitive impairment. Additionally, this review highlights the prospective application of combined multimodal neuroimaging and machine learning algorithms. By integrating readily available multidimensional clinical data, the establishment of early risk prediction models for AKI-related brain dysfunction can facilitate timely identification and precise intervention for high-risk populations. Furthermore, we elaborate the therapeutic potential of targeted anti-inflammatory strategies and natural bioactive compounds in ameliorating AKI-associated brain dysfunction. Collectively, this review provides novel insights for the development of optimized, safe and effective clinical interventions in the future.

## 1 Introduction

AKI is a common clinical syndrome characterized by a rapid (within 48 hours) decline in renal excretory function. Its etiologies are traditionally categorized into three groups: prerenal (e.g., hypovolemia, decreased cardiac output), intrinsic renal (e.g., ischemia, nephrotoxins, sepsis-induced glomerulonephritis or acute tubular necrosis), and postrenal (e.g., urinary tract obstruction) (1, 2). According to the KDIGO clinical practice guidelines, the diagnosis and staging of AKI are based on an increase in serum creatinine by ≥0.3 mg/dL within 48 hours, or to ≥1.5 times baseline within the prior 7 days, or sustained oliguria (<0.5 mL/kg/h) for at least 6 hours (1).

The incidence of AKI in intensive care units (ICUs) exceeds 50% and is associated with prolonged hospital stays and increased mortality (3). AKI not only affects the kidneys themselves but can also lead to dysfunction in distant organs. Even mild and transient AKI may indicate an increased risk of both short- and long-term adverse outcomes, including new-onset or worsening chronic kidney disease (CKD), progression to end-stage renal disease, infections and sepsis, gastrointestinal bleeding, malignancy, fracture risk, and cardiovascular events (4).

Recent clinical studies have shown that AKI can induce both acute and chronic brain dysfunction. Such as delirium, dementia, coma, depression, and stroke. The most common manifestations include delirium and dementia (5–7). Among patients with AKI in ICU, the incidence of delirium ranges from 30% to 60%, which is significantly higher than that in patients without AKI. Moreover, within 1–5 years after AKI, the risk of dementia increases by 49%–64% compared with individuals without AKI, while the risk of mild cognitive impairment (MCI) increases two-to three-fold. Evidence indicates that AKI patients who develop delirium have an ICU mortality rate of approximately 40% – 55%, whereas those with cognitive impairment experience nearly a twofold higher 1-year all-cause mortality compared with patients without cognitive impairment (8, 9). In addition, AKI accompanied by brain dysfunction substantially increases healthcare and societal costs (10, 11).

Based on existing clinical and experimental evidence, this review systematically summarizes the pathophysiological mechanisms and potential therapeutic strategies of AKI-associated brain dysfunction, with the aim of providing a theoretical basis for the early identification and clinical intervention of neurological impairment in patients with AKI, as well as offering references for future research in this field.

## 2 Acute kidney injury patients exhibit marked cerebral dysfunction

Previous studies have shown that AKI is closely associated with an increased risk of delirium and coma in critically ill patients. Siew et al. found that the occurrence of AKI was associated with a higher risk of delirium and coma, and the strength of this association increased with the severity of AKI (5). Similarly, a study by Jäckel et al. reported that among patients with stage 1 AKI, delirium occurred in 105 out of 196 cases (53.6%), while the incidence of delirium increased further in patients with stage 2 and stage 3 AKI (12). More recently, a machine learning –based study constructed early prediction models for sepsis-associated delirium (SAD) in ICU patients with sepsis-associated AKI (SA-AKI) using the MIMIC-IV (Medical Information Mart for Intensive Care IV) database and the eICU Collaborative Research Database (eICU-CRD). Multivariable logistic regression analysis demonstrated that SA-AKI is an independent risk factor for SAD in patients with sepsis, and the incidence of SAD increases progressively with advancing AKI stage (13). Delirium is not only associated with short-term adverse outcomes—such as higher mortality, increased rates of endotracheal intubation, longer hospital stays, and higher hospitalization costs (10, 12), but is also significantly associated with long-term cognitive impairment (14).

Growing evidence suggests that AKI may be an important risk factor for subsequent cognitive decline and dementia, particularly in older adults. In a cohort study of 8, 148 elderly individuals at high cardiovascular risk, multivariable-adjusted Cox regression analysis demonstrated that the development of AKI was associated with a higher likelihood of early symptoms suggestive of dementia and an increased risk of the composite outcome of dementia and MCI (15). These findings suggest that AKI may exert an effect during the early stages of cognitive decline, consistent with results from previous studies. Another study using a Cox regression model further characterized the association between AKI features and dementia risk (16). The results showed that patients who had experienced AKI had a 49% higher risk of developing dementia. Moreover, this risk increased with the severity of AKI: the risk increased by 1.45-fold after stage 1 AKI and rose further to 1.61-fold after stage 2–3 AKI. In addition, hospital-acquired AKI showed a stronger association with dementia than community-acquired AKI, suggesting that AKI arising in different clinical contexts may affect brain function through distinct mechanisms (16). Regarding dementia subtypes, Alzheimer’s disease (AD) was the most common; however, the risk of all dementia subtypes increased significantly following AKI, with the most pronounced increases observed in dementia with Lewy bodies and Parkinson ‘s disease dementia (16). These findings have been further supported by analyses of multiple databases, including the Chinese Renal Data System (CRDS) and the UK Biobank (UKB), which confirmed the robust association between AKI and an increased risk of dementia (17). Dementia has become the seventh leading cause of death worldwide, and the disease burden continues to increase with population aging (11). As a potentially modifiable risk factor, the association between AKI and cognitive decline carries important clinical and public health implications.

From a clinical perspective, elucidating the key mechanisms underlying AKI-induced brain dysfunction and developing individualized therapeutic strategies are crucial for early screening and timely intervention. Such efforts may not only reduce the occurrence of related adverse events but also significantly improve treatment success rates and the overall quality of life in patients with AKI.

## 3 Mechanisms of AKI-induced brain dysfunction

Before delving into the pathological crosstalk, it is important to recognize that the kidney possesses intrinsic compensatory mechanisms during the progression of AKI. These include hyperfiltration by surviving nephrons (18), metabolic adaptation (e.g., shifting from fatty acid oxidation to glycolysis-dominant energy metabolism in tubular cells) (19), and activation of tubular repair and regeneration programs (e.g., dedifferentiation and proliferation of viable tubular epithelial cells) (20). However, when the initial insult is severe or sustained, these compensatory responses become overwhelmed or maladaptive, leading to the accumulation of uremic toxins, sustained inflammation, and the distant organ injury — including brain dysfunction — that is the focus of this review (21).

**Figure 1:**
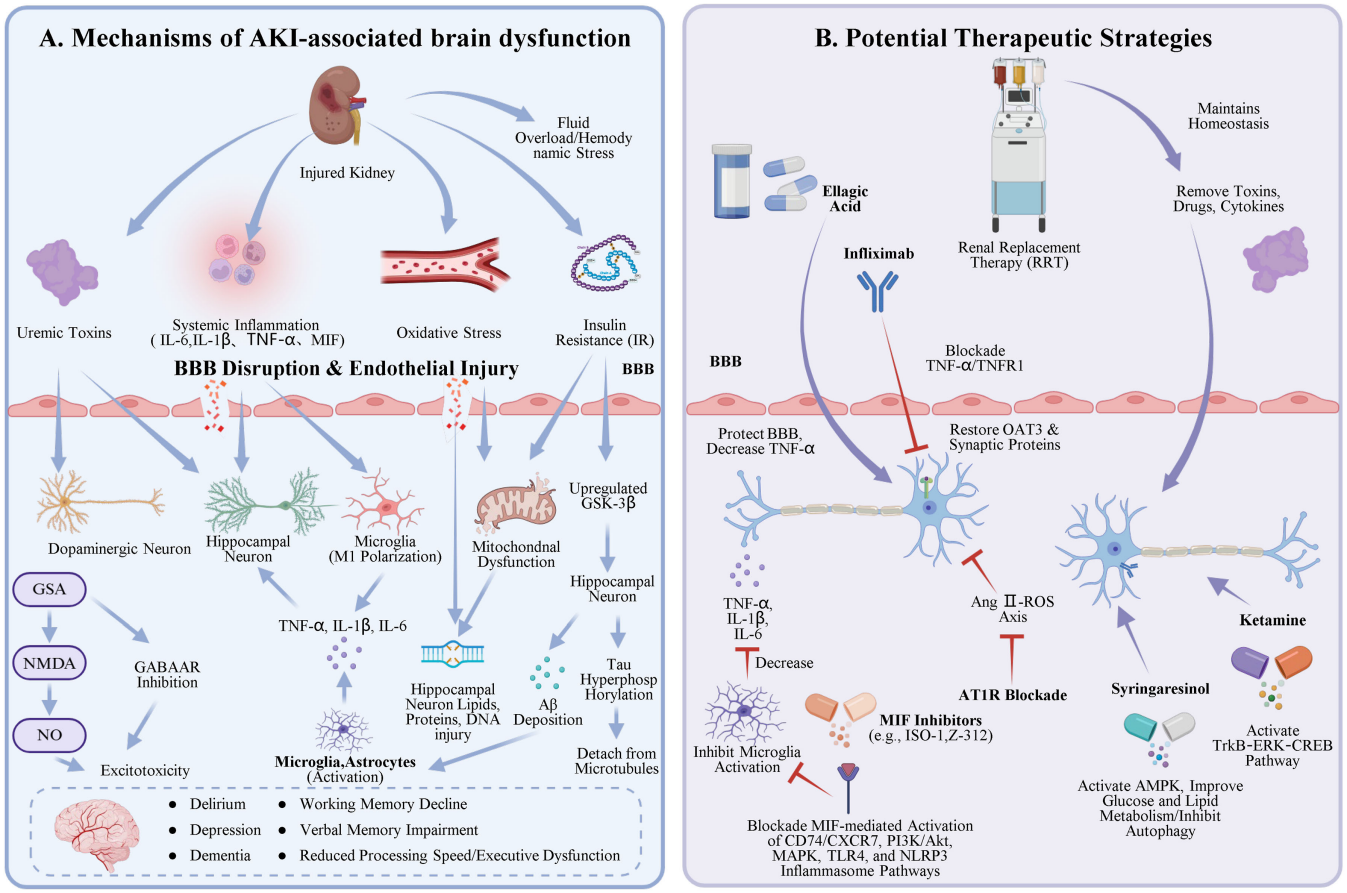
Mechanisms of acute kidney injury (AKI)-associated brain dysfunction and potential therapeutic strategies. **(A)** Systemic insults from the injured kidney disrupt blood-brain barrier integrity, triggering neuronal injury, microglial activation, mitochondrial dysfunction and tau/Aβ pathology, which ultimately lead to multiple neurocognitive and neuropsychiatric deficits. **(B)** Renal replacement therapy and targeted pharmacological agents exert neuroprotective effects via anti-inflammation, blood-brain barrier protection, synaptic restoration and metabolic regulation. Aβ, amyloid-β; AKI, acute kidney injury; AMPK, AMP-activated protein kinase; AT1R, angiotensin II type 1 receptor; BBB, blood-brain barrier; CREB, cAMP response element-binding protein; ERK, extracellular signal-regulated kinase; GABAAR, γ-aminobutyric acid type A receptor; GSK-3β, glycogen synthase kinase-3β; GSA, guanidinosuccinic acid; IL, interleukin; IR, insulin resistance; MIF, macrophage migration inhibitory factor; NMDA, N-methyl-D-aspartate; NO, nitric oxide; OAT3, organic anion transporter 3; ROS, reactive oxygen species; RRT, renal replacement therapy; TNF-α, tumor necrosis factor-α; TrkB, tropomyosin receptor kinase B.

As summarized in **Figure 1A**, AKI-associated brain dysfunction may arise from multiple interconnected mechanisms, including uremic toxin and drug accumulation, systemic inflammation and oxidative stress, BBB disruption, metabolic disturbances, dopaminergic pathway dysfunction, and IR.

### 3.1 Uremic toxins, drug accumulation, and inflammatory oxidative stress

Impaired renal excretory function during AKI can lead to the accumulation of uremic toxins, which represents one of the key mechanisms underlying cognitive impairment (22). Elevated levels of several endogenous compounds, including uric acid, indoxyl sulfate (IS), homocysteine (Hcy), parathyroid hormone (PTH), and guanidino compounds, have been closely associated with declines in working memory, verbal memory, and processing speed (23–27). Uric acid may contribute to cerebral white matter atrophy and impairments in information processing and executive function through oxidative stress and cerebrovascular injury, thereby increasing the risk of vascular dementia (24). In contrast, IS can induce inflammation and blood–brain barrier (BBB) disruption (28). Among guanidino compounds, guanidinosuccinic acid (GSA) exhibits dual neurotoxicity: it competitively antagonizes γ-aminobutyric acid type A receptors (GABA_A_Rs), thereby disrupting the excitatory/inhibitory balance, while also activating N-methyl-D-aspartate (NMDA) receptors, which results in excessive nitric oxide (NO) production and excitotoxicity. Asymmetric dimethylarginine (ADMA), by inhibiting endothelial nitric oxide synthase (eNOS), may induce vasoconstriction and reduce cerebral perfusion (29–31). In addition, the accumulation of multiple toxins may impair BBB tight junctions and aquaporin function, thereby facilitating the entry of peripheral toxic substances into the brain and promoting neuronal edema (22). Experimental studies in rat models of AKI have shown that uremic toxins significantly suppress the metabolic activity of midbrain dopaminergic neurons and impair motor coordination (24, 32).

Beyond toxin accumulation, another important contributor to delirium in patients with AKI is the reduced clearance of commonly used ICU medications (33, 34). β-Lactam antibiotics can interfere with GABA_A_Rs-mediated inhibitory neurotransmission through several mechanisms, thereby inducing central excitatory neurotoxicity (35, 36) Among these agents, cefepime-associated neurotoxicity is particularly prominent; its risk has been reported to be approximately tenfold higher than that of meropenem, with an incidence of up to 15% among ICU patients receiving cefepime (37). Consistent with clinical case reports, patients with AKI appear to be highly susceptible to central nervous system adverse effects after cefepime exposure; therefore, cefepime should be avoided when appropriate alternatives are available, or used only with renal dose adjustment and close neurological monitoring (38). Studies have shown that patients who develop delirium have, on average, 5.2 predisposing factors and 3.0 precipitating factors, among which medications, particularly opioids, are important triggers (39). Compared with patients who do not receive opioids, those treated with tramadol or pethidine have an increased risk of delirium (40). Among opioids, pethidine and morphine are prone to accumulation because of renal excretion, thereby increasing the risk of delirium (5). By contrast, fentanyl is associated with a lower risk of delirium and is recommended as the preferred analgesic in this setting (8).

Inflammatory cascades and oxidative stress play pivotal roles in the pathogenesis of AKI (41, 42). Owing to its high metabolic demand and marked sensitivity to inflammation, the hippocampus is a major target of AKI-associated systemic injury (43). After AKI, injured renal tubular epithelial cells release large amounts of pro-inflammatory cytokines, including tumor necrosis factor-α (TNF-α), interleukin-6 (IL-6), and interleukin-1β (IL-1β), as well as chemokines such as C-X-C motif chemokine ligand 1 (CXCL1) and macrophage inflammatory protein-2 (MIP-2) (44). Clinical studies have shown that elevated postoperative circulating levels of soluble tumor necrosis factor receptor 1 (sTNFR-1) are significantly associated with a 35% higher risk of cognitive decline during a 3-year follow-up period (45).

These peripheral inflammatory mediators can enter brain tissue through a disrupted BBB, induce microglial activation, and promote polarization toward a pro-inflammatory M1 phenotype; activated microglia further secrete IL-1β and TNF-α, thereby forming a vicious cycle of inflammatory activation and tissue injury (46). Activated microglia may further exacerbate BBB disruption and directly injure hippocampal neurons through apoptotic signaling pathways (46). In addition, soluble inflammatory mediators can be locally generated in the hippocampus and may directly compromise BBB integrity. Once BBB permeability is increased, peripheral inflammatory mediators, metabolic waste products, and toxic substances can extensively infiltrate the hippocampus, inducing local edema and triggering inflammatory cascades (24, 43, 47, 48). Animal studies have demonstrated that Evans blue dye concentrations in the brain tissue of AKI mice are significantly higher than those in control mice, indicating increased vascular permeability and BBB dysfunction during AKI (49). Functional magnetic resonance imaging (fMRI) showed that the amplitude of low-frequency fluctuation (ALFF) in the hippocampus was significantly reduced in AKI model mice, and changes in ALFF were significantly correlated with microglial abundance (46). Moreover, sustained activation of microglia and astrocytes plays a critical role in various neurological and neurodegenerative conditions, including ischemic stroke, epilepsy, and AD, suggesting that may contribute to long-term brain dysfunction through similar mechanisms (50).

Oxidative stress acts synergistically with inflammation to exacerbate tissue injury. AKI increases circulating levels of reactive oxygen species (ROS) and reactive nitrogen species (RNS), leading to oxidative damage to lipids, proteins, and DNA in hippocampal neurons. This oxidative damage can further trigger molecular cascades that increase BBB permeability, promote cerebral edema, and aggravate ischemia–reperfusion injury (51, 52). In addition, peripheral angiotensin II (Ang II) may enter the central nervous system through circumventricular organs, which lack a complete BBB, or through a disrupted BBB; subsequently, Ang II can act on angiotensin II type 1 receptors (AT1Rs), increase oxidative stress in the cerebellum, and indirectly affect hippocampus-dependent cognitive function (53) Together, toxin accumulation, drug exposure, systemic inflammation, and oxidative stress may mutually reinforce one another and collectively constitute core mechanisms underlying AKI-associated cognitive impairment.

### 3.2 Other mechanisms: volume overload, kidney–brain vascular injury, and metabolic disturbances

Volume overload: Fluid overload occurs in approximately 40% of patients in ICU and is closely associated with AKI (54–56) When defined as a ≥10% increase in body weight from baseline, fluid overload significantly prolongs the duration of acute brain dysfunction (OR = 2.16) and independently increases the risk of delirium (54, 57). A recent study of patients with acute stroke showed that an average daily fluid intake of >1 L during the first 5 days of ICU admission was significantly associated with an increased risk of poor long-term functional and neurological outcomes (58). In the setting of AKI, BBB integrity is often compromised, and excessive fluid accumulation may directly aggravate cerebral edema, initiating a vicious cycle of increased intracranial pressure and inadequate cerebral perfusion; it may also activate systemic inflammatory responses, further damage the vascular endothelium, disrupt BBB integrity, impair cerebrovascular autoregulation, reduce the brain’s ability to compensate for blood pressure fluctuations, and increase the risk of secondary ischemic or hemorrhagic events (58). Therefore, appropriate fluid management is essential not only for preserving renal function and preventing further kidney injury but also for maintaining adequate cerebral perfusion and reducing the risk of brain dysfunction.

Kidney–brain vascular injury axis: AKI may independently affect long-term stroke risk and mortality (7). The prevalence of severe cognitive impairment among patients undergoing hemodialysis reaches 30%–40% (22). Clinical data indicate that AKI occurs in 14% of hospitalized patients with ischemic stroke and 21% of those with intracerebral hemorrhage, and that AKI significantly increases the risk of 3-month post-stroke mortality (59). AKI often co-occurs with systemic hypotension due to sepsis, intravascular fluid shifts, and renal replacement therapy (RRT). These acute blood pressure fluctuations directly threaten cerebral perfusion, especially as sepsis and inflammation frequently impair cerebral autoregulation (7, 60). Recurrent hypotensive events can reduce cerebral blood flow below the lower limit of autoregulation, thereby triggering cerebral hypoperfusion, ischemic neuronal damage, acute delirium, and subsequent long-term cognitive impairment (60). In addition, AKI induces a series of nontraditional cerebrovascular risk factors, including endothelial dysfunction, oxidative stress, and hyperhomocysteinemia, which may simultaneously accelerate renal and cerebral atherosclerosis and contribute to a pathological state characterized by kidney–brain vascular injury crosstalk (7). Moreover, AKI is frequently accompanied by coagulation abnormalities, including consumption of coagulation factors, a hyperfibrinolytic state see (61), and microthrombus formation, which further increase the risk of thrombotic and hemorrhagic stroke (62).

Metabolic abnormalities and dopaminergic pathway dysfunction: AKI can suppress dopamine turnover in the brain. Animal studies have shown that dopamine metabolism is significantly reduced in the brains of rats with AKI, whereas norepinephrine is not substantially affected (32). Clinically, patients with uremia frequently develop restless legs syndrome, and levodopa and dopamine receptor agonists can effectively alleviate symptoms. These findings suggest that dopaminergic suppression is an important mechanism underlying AKI-associated neurological dysfunction (32). During AKI, the accumulation of toxic metabolites may directly impair the dopaminergic system. For example, aluminum may induce brain dysfunction by interfering with neurotransmitter signaling, including dopaminergic signaling. The accumulation of typical uremic toxins, such as IS and p-cresyl sulfate, may induce ROS generation and mitochondrial dysfunction, leading to apoptosis of dopaminergic neurons in the mesencephalic nigrostriatal pathway and reducing dopamine synthesis and release (24). In AKI mice, circulating C16-ceramide levels are significantly elevated and accompanied by depression-like behaviors; meanwhile, levels of dopamine 4-sulfate, which is primarily secreted by neuronal cells, are reduced, suggesting neurological injury (63). Elevated C16-ceramide levels are closely associated with neuronal apoptosis, oxidative stress, and pro-inflammatory gene expression and are also a common feature of neurodegenerative diseases such as AD (64). This alteration may partially explain the pathogenesis of AKI-associated depression (63). Overall, AKI may contribute to cognitive impairments, including deficits in memory and learning, as well as abnormalities in emotional regulation, by suppressing dopaminergic excitatory transmission and disrupting metabolic homeostasis.

### 3.3 Insulin resistance

The kidney plays a crucial role in maintaining glucose homeostasis, contributing to approximately 30% of daily gluconeogenesis and accounting for nearly 50% of systemic insulin metabolism and clearance (65, 66). IR is commonly observed in patients with AKI, and this pathological state is closely associated with the severity of renal dysfunction, levels of inflammatory markers such as C-reactive protein (CRP) and interleukin-6 (IL-6), and the accumulation of uremic toxins. Compared with healthy controls, patients with AKI exhibit significantly elevated serum creatinine, urea, uric acid, CRP, IL-6, and homeostatic model assessment of IR (HOMA-IR), indicating that IR is closely linked to the pathophysiological progression of AKI (67). Insulin-like growth factor binding protein 7 (IGFBP7) are key regulatory molecules involved in the pathogenesis and progression of IR. They are significantly upregulated in both serum and urine of patients with AKI and mouse AKI models, and their expression levels are positively correlated with the severity of renal dysfunction (68). IR has now been well established as a core pathophysiological factor driving the development, progression, and deterioration of AKI (69).

The triglyceride–glucose (TyG) index, a simple and widely used surrogate marker for assessing IR 20 (70), has been shown to be closely associated with SA-AKI and length of hospital stay (71, 72), and in recent years has also been linked to cognitive impairment. A multicenter study conducted by Xiaxuan Huang and colleagues demonstrated that among individuals aged 65 years and older, the probability of delirium increased significantly when the TyG index exceeded a threshold of 8.912 (73, 74). Notably, in patients with severe AKI, Wenhui Zhang and colleagues applied a Cox proportional hazards model and found that the TyG index, treated as a continuous variable, was a significant risk factor for delirium. In the unadjusted model, the hazard ratio (HR) was 1.24 (95% CI: 1.10– 1.41, P < 0.001), and after full adjustment for confounding factors, the HR remained significant at 1.20 (95% CI: 1.04– 1.39, P < 0.05) (75). Recent evidence showed that both peripheral and central IR play a role in the pathophysiology, clinical presentation, and management of neuropsychiatric disorders like Cognitive Impairment/Dementia, Depression, and Schizophrenia (76).

The mechanisms by which IR contributes to cognitive dysfunction can be systematically explained from four interrelated dimensions — neuronal injury, abnormal protein metabolism, neuroinflammation, and genetic susceptibility — with substantial synergistic interactions among these mechanisms.

Local IR within the brain can enhance the activity of glycogen synthase kinase-3 β (GSK-3β) (77), thereby inducing excessive phosphorylation of Tau protein. As a microtubule-associated protein in neurons, the physiological phosphorylation state of Tau is essential for maintaining microtubule stability and axonal transport. However, hyperphosphorylation causes Tau to detach from microtubules, directly disrupting neuronal structure and signal transmission and ultimately leading to cognitive impairment (78). Animal studies further support this mechanism. In fructose-induced IR models, significantly increased levels of phosphorylated Tau have been observed in mouse brain tissue (79).

Similarly, mice with high-fat-diet – induced IR not only exhibit typical metabolic features of IR, including elevated serum glucose, triglyceride, cholesterol, and insulin levels, but also show marked oxidative stress and mitochondrial dysfunction. These animals display significantly impaired spatial learning and memory, suggesting that IR may exacerbate cognitive deficits through the combined effects of the “GSK-3 β–Tau phosphorylation “ pathway and disturbances in energy metabolism (77, 80).

During the early stages of IR, hyperglycemia and disrupted insulin signaling bidirectionally regulate amyloid-β (Aβ) metabolism. On the one hand, hyperglycemia and IR promote the formation of Aβ oligomers in the brain, facilitating abnormal Aβ deposition and disrupting neuronal membrane integrity (77, 81). On the other hand, insulin-degrading enzyme (IDE), a key enzyme responsible for degrading Aβ monomers in the cerebrospinal fluid, is influenced by circulating insulin levels. Under conditions of IR, elevated insulin concentrations cause IDE to preferentially degrade insulin rather than Aβ, thereby impairing Aβ clearance and further aggravating Aβ accumulation (82). The abnormal accumulation of Aβ subsequently activates microglia and astrocytes, leading to the release of pro-inflammatory cytokines and the generation of reactive oxygen species. This neuroinflammatory response exacerbates neuronal dysfunction, synaptic loss, and further Aβ deposition. Prolonged exposure to oxidative stress damages both the structure and function of the hippocampus—a critical brain region for learning and memory—thereby directly impairing the acquisition of new information and accelerating cognitive decline (83, 84).

Patients with IR exhibit a significantly higher frequency of the apolipoprotein E (ApoE) ϵ4 allele, which has been closely associated with cognitive decline (75). Previous studies have shown that, compared with individuals without IR and without the ApoE ϵ4 allele, patients with type 2 diabetes who carry the ApoE ϵ4 allele have a markedly increased risk of developing Alzheimer ‘s disease (AD), with a relative risk (RR) of 5.5 (95% CI: 2.2 – 13.7). Interactions between ApoE and Tau further exacerbate Tau-mediated neurodegeneration and Tau-related pathology. Meanwhile, individuals carrying the ApoE ϵ4 allele demonstrate increased blood – brain barrier permeability (78), a greater number of neuroinflammatory plaques in the hippocampus, significantly increased neurofibrillary tangles in both the cortex and hippocampus, and a markedly elevated risk of cerebral amyloid angiopathy (85). Recent studies further confirm that the occurrence of dementia is significantly associated with diabetes (an IR-related disease) and the presence of the ApoE ϵ4 allele. Notably, carriage of the ApoE ϵ4 allele increases the risk of AD by approximately three-to four-fold, highlighting the important modulatory role of genetic background in IR-related cognitive impairment (86).

## 4 Potential therapeutic strategies

### 4.1 Targeting TNF-α/TNFR1 and MIF for anti-inflammatory treatment: promising therapeutic strategies for AKI-associated brain dysfunction

TNFR1 is widely expressed on the surface of nearly all nucleated cells and mediates the pro-inflammatory and pro-apoptotic effects of TNF-α. Studies have shown that AKI is associated with lower scores on the Modified Mini-Mental State Examination (3MS), while soluble tumor necrosis factor receptor-1 (sTNFR- 1) largely mediates the risk of long-term cognitive impairment (45). More importantly, blockade of the TNFR1 signaling pathway through genetic manipulation or pharmacological intervention has been shown to effectively delay cognitive decline in mouse models of AD (87).The blood–cerebrospinal fluid barrier is formed by a monolayer of choroid plexus epithelial (CPE) cells. Relevant studies have demonstrated that TNF acts as a major upstream inflammatory mediator in the choroid plexus tissue of patients with AD. Activation of TNFR1 leads to morphological damage of CPE cells in AD models, whereas deletion of TNFR1 alleviates neuroinflammation and prevents dysfunction of the blood – cerebrospinal fluid barrier (87). In addition, genetic deletion of TNFR1 suppresses A β production, reduces the formation of A β plaques in the brain, attenuates neuronal injury in mice, and markedly improves memory impairment (88).

Experiments in cisplatin-induced AKI animal models demonstrated that both rats and mice developed four concurrent pathological changes: significantly reduced expression of organic anion transporter 3 (OAT3), synaptophysin, and microtubule-associated protein 2 (MAP2), suppressed locomotor and exploratory behaviors, and increased accumulation of uremic toxins in the brain. Further mechanistic studies confirmed that AKI causes a rapid decline in renal function, leading to marked accumulation of anionic uremic toxins such as IS in the circulation and brain. This accumulation activates brain macrophages and microglia, triggering the IS- TNF-α signaling axis. This axis exerts dual detrimental effects: First, it markedly suppresses the expression of synaptophysin and MAP2. MAP2 plays an important role in maintaining neuronal plasticity, whereas reduced synaptophysin levels and decreased dendritic spine density have been associated with multiple neuropsychiatric disorders, including intellectual disability, depression, and schizophrenia (28). Second, this pathway can downregulate OAT3 expression in the brain. OAT3 is primarily localized to the basolateral membrane of brain microvascular endothelial cells and the apical membrane of choroid plexus epithelial cells, where it mediates the transport of IS from the brain to the peripheral circulation and participates in the efflux of neurotransmitter metabolites and exogenous drugs (89). Most anionic uremic toxins exert inhibitory effects on organic anion transporters (OATs). The uremic milieu may simultaneously reduce OAT3 protein expression and impair its transport function, resulting in abnormal intracerebral accumulation of neurotransmitter metabolites and drugs. This process may ultimately establish a vicious cascade characterized by toxin accumulation, OAT3 downregulation, and further toxin retention, thereby contributing to the onset and progression of brain dysfunction. Following specific blockade of TNF-α with infliximab, central nervous system behavioral abnormalities were significantly ameliorated in AKI animals treated with IS or TNF-α. The downregulation of synaptophysin, MAP2, and OAT3 protein expression was completely reversed, and the abnormally elevated levels of uremic toxins in the brain were markedly attenuated (28). However, no consensus has been reached regarding the expression pattern and biological function of OAT3 in human tissues, and this uncertainty represents a major limitation of the study. Therefore, whether targeted TNF-α blockade can effectively ameliorate brain dysfunction in patients with AKI requires further investigation.

Macrophage migration inhibitory factor (MIF) is a multifunctional cytokine with intrinsic topoisomerase activity that extensively regulates multiple core biological processes, including cell proliferation, metabolism, migration, differentiation, and programmed cell death (90). Clinical studies have demonstrated that plasma and urinary MIF levels are significantly elevated in patients with AKI, and its expression abundance is positively correlated with the severity of renal injury, systemic oxidative stress, and endothelial dysfunction (91). Experimental studies have further validated the regulatory role of MIF: synchronized upregulation of MIF and pyroptosis pathway-related proteins was observed in renal tissues of SA-AKI mouse models established by cecal ligation and puncture (CLP) and in human renal tubular epithelial HK-2 cell models injured by lipopolysaccharide (LPS) (90). MIF knockdown significantly inhibited NLRP3 inflammasome activation and thereby attenuated renal tubular epithelial cell pyroptosis in HK-2 cells, while it markedly reduced NLRP3 inflammasome expression and alleviated dopaminergic neuron damage in microglia (90, 92).

Furthermore, MIF can activate microglia and astrocytes by recruiting various inflammatory mediators, thereby mediating neuroinflammatory responses in the central nervous system (CNS) (93). Therefore, MIF-targeted drugs hold promise as potential therapeutic strategies for cognitive impairment disorders. At the molecular level, elevated circulating MIF can cross the BBB. By binding to the CD74/CXCR7 receptor complex, MIF activates the phosphatidylinositol 3-kinase/protein kinase B (PI3K/Akt) signaling pathway in various diseases including ischemic stroke, promoting the release of proinflammatory cytokines such as IL- 1 β, IL-6, and TNF- α. In cognitive impairment disorders, activation of this pathway promotes the abnormal deposition of A β and tau proteins. Additionally, activation of the PI3K/Akt signaling pathway enables MIF to function as a chemokine, recruiting immune cells including microglia and thereby triggering neuroinflammatory responses (93).

MIF-targeted therapeutic strategies (e.g., ISO- 1 and Z-312) have demonstrated favorable renal and neuroprotective effects in animal models, making them promising potential therapeutic approaches for AKI and its associated cognitive impairment (90, 92–95). Specifically, in the rat model of acute hemorrhagic shock, ISO-1 mitigates kidney injury by inhibiting the activation of NF-κB and NLRP3 inflammasome pathways (96), and improves neurological outcomes in rodent and cellular models of diffuse axonal injury, ischemic stroke, cognitive impairment and Parkinson’s disease (93).The novel MIF inhibitor Z-312 significantly attenuates microglial activation and neuronal damage by blocking the MAPK pathway 31. Activation of the Toll-like receptor (TLR) signaling pathway exacerbates cognitive decline, and this pathway can be induced by Aβ plaques, further aggravating neuroinflammation and neuronal injury in AD (97). Phosphodiesterase inhibitors (PDEs) downregulate the expression levels of MIF and TLR4, exerting neuroprotective effects against neurodegenerative diseases (93, 98).

The consistent preclinical findings that pharmacological inhibition of the TNFR1/TNF-α signaling pathway and MIF effectively alleviates AKI-associated brain dysfunction by suppressing central nervous system inflammation provide a compelling scientific rationale for advancing these targeted anti-inflammatory approaches into early-phase clinical trials.

### 4.2 Natural bioactive compounds for improving cognitive impairment

Ellagic acid (EA) is a natural polyphenolic compound widely found in many soft- and hard-shelled fruits as well as various plant extracts, and exhibits antioxidant, antimicrobial, anti-inflammatory, and anticancer properties (49). In addition, EA has demonstrated neuroprotective effects and has been shown to improve cognitive function—particularly learning and memory—in rat models of neurodegenerative diseases (99). Experimental data indicate that, compared with AKI mice without EA intervention, increasing concentrations of EA are associated with a significant reduction in TNF-α expression in the hippocampal tissue of model mice. Meanwhile, the population spike amplitude in the hippocampal region is markedly enhanced, BBB function is improved, and the learning and memory abilities of the mice are correspondingly enhanced. Disruption of BBB permeability induced by AKI allows cytokines to infiltrate the brain, leading to cerebral edema and inflammatory responses. Furthermore, cytokine-mediated activation of microglia exacerbates BBB damage and contributes to neurological dysfunction. Owing to its anti-inflammatory properties, EA may alleviate these pathological changes and therefore represents a potential strategy for mitigating AKI-related brain injury and delaying disease progression (49).

Syringaresinol (SYR) is a major lignan compound found in cinnamon and possesses a wide range of pharmacological activities, including antioxidant, anti-inflammatory, anti-aging, antidiabetic, antimicrobial, and cardioprotective effects (100). Experimental studies have shown that SYR exerts both metabolic and neuroprotective effects in diabetic – Alzheimer ‘s disease (DM – AD) model mice. Specifically, SYR not only improves systemic metabolic dysfunction, enhances liver function, and ameliorates cognitive performance, but also alleviates oxidative stress and neuroinflammation. Molecular experiments, molecular docking analyses, and pharmacological inhibition studies have demonstrated that the core mechanism underlying these effects involves the activation of AMP-activated protein kinase (AMPK). Activation of AMPK has been closely associated with improvements in cognitive decline and attenuation of AD–related pathological features. This kinase exerts neuroprotective effects by regulating autophagy, reducing hyperphosphorylation of tau protein, and suppressing neuroinflammatory signaling pathways (100).In addition, studies have shown that IR is significantly associated with a higher incidence of delirium in patients with severe AKI (75). IR can impair AMPK activity, leading to disturbances in lipid and glucose metabolism as well as mitochondrial dysfunction (80). Taken together, SYR, as a potent natural AMPK activator with significant neuroprotective properties, represents a promising therapeutic candidate for the development of novel treatments targeting AKI-associated cognitive impairment.

### 4.3 Other pharmacological interventions for AKI-induced brain dysfunction

Accumulation of uremic toxins in patients with AKI can activate the renin–angiotensin system (RAS) and upregulate angiotensin II type 1 receptor (AT1R) expression. Meanwhile, systemic vasodilation triggered by cytokines or endotoxins may enhance sympathetic excitability, further elevating angiotensin II (Ang II) levels (53, 101). Experimental studies in uremic mouse models have confirmed a marked increase in Ang II expression within the brain, and systemic administration of Ang II receptor antagonists can effectively suppress this phenomenon (102).The inflammatory cascade induced by AKI disrupts blood – brain barrier (BBB) integrity, facilitating the entry of peripheral Ang II into the brain. Ang II subsequently activates NADPH oxidase via AT1R, leading to excessive reactive oxygen species (ROS) production and oxidative stress in the brain (53). Oxidative stress and inflammation have been implicated in cognitive impairment (46). Experimental evidence further suggests that losartan not only decreases serum creatinine and blood urea nitrogen (BUN) levels in AKI mice, thereby improving renal function, but also reduces brain Ang II levels, attenuates cerebral oxidative stress through angiotensin receptor blockade, and ultimately ameliorates spatial memory deficits in uremic mice (53). Future clinical trials targeting AT1R are warranted to validate the therapeutic potential of these agents for cognitive impairment associated with uremic encephalopathy.

Ketamine is an anesthetic agent with anti-inflammatory and antioxidant properties, and previous studies have suggested that it may exert antidepressant effects in patients with depression (103). In a cisplatin-induced AKI mouse model, animals exhibit depression-like phenotypes, including reduced sucrose preference, decreased locomotor activity, and prolonged immobility time in the forced swim test (FST). Western blot analyses further reveal downregulation of synapse-associated proteins in the prefrontal cortex (e.g., postsynaptic density protein 95 and glutamate receptor A1), resulting in synaptic structural damage and weakened excitatory neurotransmission. Ketamine treatment not only reduces BUN and creatinine levels but also alleviates depression-like behaviors in AKI mice (63). Notably, the TrkB inhibitor ANA- 12 abolishes the beneficial effects of ketamine. Combined with western blot findings, these results suggest that ketamine may mitigate renal injury and depression-like symptoms by activating TrkB and its downstream extracellular signal-regulated kinase (ERK)– cAMP response element-binding protein (CREB) signaling pathway (63, 104).

### 4.4 Renal replacement therapy

Renal replacement therapy (RRT) provides an important supportive intervention for patients with severe AKI and multiple organ failure (MOF). Studies have shown that among patients who did not receive RRT, after adjustment for relevant covariates, each 1 mg/dl increase in the daily peak serum creatinine level was associated with a significantly higher risk of delirium (OR 1.35, 95% CI 1.18–1.55) and coma (OR 1.44, 95% CI 1.20–1.74). In contrast, among patients receiving RRT, no significant association was observed between the daily peak serum creatinine level and the occurrence of delirium (OR 1.07, 95% CI 0.87–1.31) or coma (OR 1.16, 95% CI 0.94–1.44). These findings suggest that RRT may alter the relationship between acute changes in serum creatinine and acute brain dysfunction (5).The potential mechanisms underlying this effect may include the ability of RRT to remove potentially neurotoxic substances, such as sedatives, antibiotics, and other metabolic products (8, 33). In addition, RRT may eliminate or modulate inflammatory mediators and help restore immune homeostasis, thereby mitigating the impact of AKI on the brain (105). However, patients with AKI are prone to hypotension during dialysis, which may lead to reduced cerebral perfusion. Furthermore, dialysate may impair the anti-inflammatory and clearance functions of neutrophils, indirectly promoting thrombosis and increasing the risk of stroke (7). Therefore, although RRT may theoretically alleviate AKI-associated brain dysfunction through mechanisms such as the removal of neurotoxic substances and regulation of inflammatory responses, current evidence remains insufficient to draw definitive conclusions. Nevertheless, RRT plays an indispensable role in correcting uremia, maintaining internal homeostasis, and supporting multiple organ function. Future studies are needed to clarify the mechanisms by which RRT influences brain function and to determine the optimal timing, modality, and dosage of RRT in order to protect renal function while minimizing the risk of neurological complications.

Potential therapeutic strategies for AKI-associated brain dysfunction mainly target neuroinflammation, uremic toxin accumulation, BBB disruption, oxidative stress, metabolic dysfunction, synaptic injury, and systemic homeostatic imbalance. These strategies are summarized and illustrated in the **Figure 1B** and **Table 1**.

**Table 1:** Potential therapeutic strategies.

Therapeutic strategy	Representative agents/interventions	Key mechanisms	Potential neuroprotective effects	Current evidence/limitations
TNF-α/TNFR1 pathway inhibition	TNFR1 blockade, infliximab	Inhibits TNF-α/TNFR1-mediated inflammation and apoptosis; reduces microglial/macrophage activation; preserves blood–cerebrospinal fluid barrier integrity; restores OAT3 expression and promotes uremic toxin clearance	Reduces neuroinflammation, synaptic injury, uremic toxin accumulation, and cognitive dysfunction	Mainly supported by animal studies; clinical validation in AKI-associated brain dysfunction is needed
MIF-targeted therapy	ISO-1, Z-312, MIF inhibition	Suppresses MIF-mediated activation of CD74/CXCR7, PI3K/Akt, MAPK, TLR4, and NLRP3 inflammasome pathways; reduces pyroptosis and inflammatory cytokine release	Attenuates renal inflammation, microglial activation, neuronal injury, and cognitive impairment	Promising preclinical evidence; requires translational and clinical studies
Natural anti-inflammatory and antioxidant compounds	Ellagic acid	Reduces hippocampal TNF-α expression; protects BBB integrity; decreases cytokine infiltration and microglial activation	Improves hippocampal synaptic function, learning, and memory in AKI models	Evidence mainly from experimental models
AMPK-activating natural compounds	Syringaresinol	Activates AMPK signaling; improves metabolic dysfunction and insulin resistance; reduces oxidative stress, neuroinflammation, mitochondrial dysfunction, tau hyperphosphorylation, and impaired autophagy	May improve AKI-associated cognitive impairment, especially when metabolic dysfunction or insulin resistance is involved	Indirect evidence from diabetic–AD models; AKI-specific studies are needed
Renin–angiotensin system modulation	Losartan, AT1R antagonists	Blocks Ang II/AT1R signaling; inhibits NADPH oxidase activation; reduces ROS production, oxidative stress, and neuroinflammation	Ameliorates spatial memory deficits and cerebral oxidative injury in uremic/AKI models	Preclinical evidence is promising; clinical trials are required
Ketamine-based intervention	Ketamine	Activates TrkB–ERK–CREB signaling; restores synaptic proteins such as PSD95 and GluA1; exerts anti-inflammatory and antioxidant effects	Improves depression-like behaviors and synaptic dysfunction after AKI	Mainly based on cisplatin-induced AKI models; safety and indications require careful evaluation
Renal replacement therapy	CRRT, intermittent hemodialysis	Removes uremic toxins, neurotoxic drugs, and metabolic products; modulates inflammatory mediators; restores fluid, electrolyte, and acid–base homeostasis	May reduce delirium/coma risk by decreasing systemic toxicity and inflammatory burden	Evidence remains inconclusive; dialysis-related hypotension may reduce cerebral perfusion and increase neurological risk

## 5 Early prediction of AKI-associated brain dysfunction: from insulin resistance markers to the integration of multimodal neuroimaging and machine learning

### 5.1 Insulin resistance markers

AKI has become a major global public health issue, with approximately 13.3 million new cases reported worldwide each year (106). Currently, effective pharmacological therapies for AKI remain limited, highlighting the practical importance of identifying novel modifiable risk factors and preventing AKI-associated brain dysfunction. IR is closely associated with the occurrence and progression of AKI, and multiple studies have investigated the predictive performance of various IR-related indices for AKI.Qin et al. demonstrated that the TyG index is an independent risk factor for contrast-associated AKI in patients with type 2 diabetes mellitus (107); a cohort study by Fang et al. further showed that the TyG index also has predictive value for SA-AKI (72). Wang et al. found that when the metabolic score for insulin resistance (METS-IR) was modeled as a continuous variable, the risk of both AKI and stage III AKI increased with higher METS-IR levels, regardless of adjustment for confounders such as age, sex, race, and body mass index (BMI); however, no similar trend was observed for the TyG index or the triglyceride/high-density lipoprotein cholesterol ratio (TG/HDL-C). This study further confirmed that only METS-IR was independently associated with the occurrence of AKI and stage III AKI, and additional analyses revealed a nonlinear increase in AKI risk with increasing METS-IR levels. Subgroup analyses indicated population heterogeneity in the predictive value of METS-IR for AKI: no statistically significant association was observed among patients with heart failure, CKD, or diabetes, and no interaction effects were detected across subgroups. Subgroup analyses for stage III AKI also suggested that METS-IR may not be suitable for assessing disease risk in patients with concomitant heart failure (108).

Another study showed that the estimated glucose disposal rate (eGDR) independently predicts AKI risk in individuals with dysglycemia (109). The eGDR integrates vascular-related parameters, such as hypertension and abdominal obesity, with metabolic parameters, such as glycated hemoglobin (HbA1c), thereby providing a more comprehensive reflection of IR-mediated multisystem pathological alterations. The study reported that lower eGDR levels indicate more severe IR and are associated with a higher risk of AKI, with a clear dose–response relationship: individuals in the highest eGDR quartile had a 62% lower risk of AKI than those in the lowest quartile. Subgroup analyses confirmed that the association between eGDR and AKI remained stable across different populations. Moreover, compared with commonly used IR markers, including the TyG index, TG/HDL-C ratio, C-reactive protein–triglyceride–glucose index (CTI), single-point insulin sensitivity estimator (SPISE), METS-IR, and lipid accumulation product (LAP), eGDR showed superior predictive performance for AKI risk (109).

Several studies have also confirmed that IR-related indices are closely associated with central nervous system injury. The MIND-China study conducted by Tian et al. found that an elevated TyG index significantly increased the risk of dementia and AD, and this association persisted even among individuals without cardiovascular disease or diabetes (110). Wang et al. demonstrated that the TyG index, triglyceride–glucose body mass index (TyG-BMI), METS-IR, and TG/HDL-C are independent risk factors for post-stroke depression and may be used for early identification of high-risk populations (111). A multicenter study by Hou et al. found that higher METS-IR levels were significantly associated with an increased risk of severe neurological deficits in patients with severe cerebral infarction (112) As noted above, Zhang et al. found that a high TyG index was closely associated with delirium in patients with severe AKI (75).

In summary, IR is common among patients with AKI, and various IR-related indices have been shown to effectively predict the risk of AKI (**Table 2**). Based on existing evidence, future studies should further explore the predictive value of different IR markers for neurological adverse events secondary to AKI, such as dementia, depression, and stroke.

**Table 2:** Potential value of insulin resistance markers.

Category	Equations/context	Main findings	Advantages	References
TyG index	ln (triglycerides [mg/dl] × glucose [mg/dl]/2)	Higher TyG is associated with contrast-associated AKI and sepsis-associated AKI	Based on routine laboratory parameters; simple to calculate, low cost, suitable for large-scale screening and bedside rapid assessment.	(71–73, 75, 107, 110, 111)
METS-IR	ln ((2 × glucose [mg/dl]) + triglycerides [mg/dl]) ×BMI [kg/m²])/(ln (HDL-C [mg/dl])	Higher METS-IR is associated with increased risk of AKI and stage III AKI	Integrates glucose, lipid, and obesity parameters; reflects metabolic disturbances more comprehensively than single indicators; provides independent predictive value for AKI risk.	(108, 111, 112)
eGDR	21.158 + (0.09 × WC) - (3.407 × HT) - (0.551 × HbA1c), where WC denotes waist circumference (cm), HT represents hypertension status (1=present; 0=absent)	Lower eGDR indicates more severe IR and higher AKI risk	Combines vascular parameters and metabolic parameters, better reflecting IR-mediated multisystem pathological changes; association remained stable across different populations.	(109)
Overall summary	TyG index, METS-IR, eGDR	IR are associated with increased risk of AKI and its severity, closely linked to AKI-related brain dysfunction	Clinically accessible and easily calculable markers enable early risk stratification for both kidney and brain complication.	(75, 106, 110–112)

### 5.2 Integration of multimodal neuroimaging and machine learning

Although risk prediction models based on clinical and biochemical indicators have shown potential utility in the early screening of AKI-associated brain dysfunction, such approaches are limited in their ability to elucidate the neuropathological mechanisms underlying AKI-associated brain dysfunction or to achieve precise phenotypic stratification (13) Multimodal neuroimaging enables noninvasive assessment of abnormalities in brain structure, function, metabolism, and perfusion, whereas machine learning is well suited to extracting predictive features from high-dimensional radiomics data. Their integration may overcome the inherent limitations of conventional assessment systems and provide new avenues for early screening and individualized precision intervention in AKI-associated brain dysfunction. **Table 3** summarizes the potential roles, advantages, and limitations of multimodal neuroimaging and machine learning approaches for AKI-associated brain dysfunction.

**Table 3:** Summary of multimodal neuroimaging and machine learning.

Technique/method	Key content and application	Advantages	Limitations	References
rs-fMRI	Detects AKI-related functional network abnormalities via resting-state BOLD signals; BET improves preprocessing.	Noninvasive assessment of brain function	Limited feasibility in ICU; affected by motion and sedation	(113, 114)
Deep learning-enhanced MRI	Applies deep learning for MRI preprocessing and feature extraction to enhance image quality and stability for.	Improves image quality and feature stability for quantitative brain assessment.	Interpretability and cross- center generalizability need validation	(114)
Radiomics + deep learning	Fuses handcrafted radiomics with deep features to build prediction models; provides a methodological framework	Integrates interpretable and deep features	Lacks AKI-specific dataset validation	(115)
EEG	Enables bedside real-time monitoring of brain activity for dynamic screening of delirium and coma in ICU patients unable to cooperate with clinical scales.	Portable, real-time, and ICU-friendly	Susceptible to noise	(113)
Deep learning-based EEG models	Uses CNN/LSTM or Vision Transformer to analyze EEG features; enables automated, stratified prediction of AKI-associated delirium risk.	Automated, bedside-applicable, and suitable for continuous monitoring	Needs large datasets and AKI-specific validation	(113, 116)
XGBoost	Integrates multimodal data (clinical, laboratory, EEG, MRI) for real-time risk stratification of AKI-associated brain dysfunction.	Strong performance with structured data	Black-box nature	(113)
SHAP/XAI1	Interprets model predictions via SHAP values; identifies key risk factors and important imaging features	Improves transparency and clinical acceptance	Explanations require further clinical validation	(113, 117)

rs-fMRI captures spontaneous fluctuations in blood oxygen level-dependent (BOLD) signals and reflects intrinsic neural activity at rest. By characterizing the topological properties of whole-brain functional connectivity, rs-fMRI studies have shown that sepsis can induce functional network disturbances in key regions, including the frontal lobe, temporal lobe, and cerebellum; abnormalities in these regions are directly associated with core clinical manifestations of delirium, such as attentional deficits and disturbances of consciousness (113). Huang et al. performed a quantitative brain function study in patients with ICU delirium using resting-state MRI preprocessed with the brain extraction tool (BET). Compared with healthy controls, patients with delirium showed significantly increased regional homogeneity (ReHo) in the pons, right hippocampus, left cerebellum, midbrain, and basal ganglia, whereas ReHo in the frontal gyrus, middle frontal gyrus, left inferior frontal gyrus, parietal lobe, and temporal lobe was significantly decreased. Fractional anisotropy (FA) was also significantly reduced in the left cerebellum, frontal lobe, left temporal lobe, corpus callosum, and left hippocampus (114). This study further demonstrated that deep learning-based optimization can improve MRI image quality, thereby strengthening the technical basis for MRI-based quantitative assessment of brain function in critically ill patients with delirium (114). Cüce et al. developed bimodal hybrid models, namely DenseASPP-RadFusion and MobileASPP-RadFusion, by integrating manually selected radiomics features with deep learning-derived features to enable automated interpretation of cerebrospinal fluid signal abnormalities on MRI in patients with central nervous system infections. The fused DenseASPP-RadFusion model achieved an accuracy of 78.57 ± 4.76% (115). Although this study focused on patients with central nervous system infections, its modeling framework combining handcrafted radiomics with automated deep learning feature extraction may provide a mature methodological reference for multimodal MRI analysis of AKI-associated brain dysfunction.

Electroencephalography (EEG) has unique advantages for real-time dynamic monitoring of brain function and is particularly suitable for critically ill patients who are unable to cooperate with bedside clinical scale assessments, such as those in coma or under deep sedation. Existing studies have shown that portable wireless EEG devices equipped with machine learning algorithms can identify high-risk patients before overt clinical symptoms of delirium appear. Some studies have constructed deep learning architectures combining convolutional neural networks (CNNs) with feedforward neural networks. In these frameworks, CNNs automatically extract spatial and waveform features from raw EEG time-domain signals, whereas long short-term memory (LSTM) networks capture temporal dependencies in EEG sequences, enabling stratified prediction of delirium risk based on abnormalities in EEG rhythms (113). Mulkey et al. applied a state-of-the-art deep learning architecture, the Vision Transformer, to rapid bedside EEG analysis using a limited number of channels and evaluated the predictive performance of a Vision Transformer-based supervised learning model for delirium in mechanically ventilated older critically ill patients. In that study, 15 heterogeneous algorithmic models were compared, and the best-performing model achieved an accuracy of >99.9% in the training set and 97% in the independent test set. Taken together, EEG combined with machine learning represents a reliable technical strategy for noninvasive, real-time, and dynamic screening of delirium (116).

Optical neuroimaging techniques, represented by near-infrared spectroscopy and diffuse optical tomography, are highly portable and offer favorable spatiotemporal resolution, making them important emerging tools for bedside brain function monitoring in the ICU. These approaches enable noninvasive real-time monitoring of cerebral tissue oxygenation, cerebral hemodynamic fluctuations, and cerebrovascular autoregulation in critically ill patients, thereby providing new observational tools for elucidating the cerebrovascular mechanisms underlying AKI-related secondary brain injury. In a focused review, Jiang et al. systematically summarized advances in optical neuroimaging in SAD, suggesting that this technology may deepen understanding of the pathophysiological mechanisms of delirium and provide theoretical support for the development of novel preventive and therapeutic targets as well as risk prediction models (118). However, the application of optical neuroimaging in SAD remains at an early stage of clinical exploration, and its predictive performance and clinical utility require further validation in large-scale prospective cohort studies.

The black-box nature of many machine learning models has long hindered their implementation in clinical practice. Explainable artificial intelligence (XAI), particularly the Shapley additive explanations (SHAP) algorithm, provides an effective solution to this challenge. SHAP quantitatively estimates the contribution of each individual feature to model predictions and provides interpretable visual outputs. For gradient-boosted tree models such as XGBoost, SHAP can generate feature-importance rankings and summary plots, in which the absolute SHAP value represents the magnitude of feature influence and the color scale indicates the direction of the association between feature values and model outcomes. Interpretable modeling can improve clinicians’ acceptance of intelligent prediction models, facilitate verification of model reasoning, and promote the translation of machine learning from experimental research into clinical decision-support tools (113). A review by Huang et al. in the field of neuropsychiatric disorders noted that embedding explainability tools such as SHAP into multimodal imaging-based artificial intelligence workflows is a key step toward the clinical translation of precision medicine (117). Accordingly, SHAP can be applied to attribution analyses of radiomics features and brain network parameters to quantitatively clarify the strength of associations between imaging abnormalities in specific brain regions and the risk of AKI-associated brain dysfunction. For example, Gao et al. further proposed a hybrid prediction model for postoperative sepsis-associated neurocognitive disorders that integrates multisource data, including the Confusion Assessment Method for the ICU (CAM-ICU), MRI and EEG radiomics parameters, and inflammatory cytokines. Using the XGBoost algorithm, this model may enable real-time risk stratification and support early diagnosis and individualized intervention (113).

Based on current evidence, a stepwise screening strategy may be useful for detecting AKI-associated brain dysfunction in hospitalized patients. An XGBoost model based on clinical indicators could first be used for initial risk stratification. Patients at moderate to high risk could then undergo bedside portable EEG to assess abnormalities in brain electrical activity. For high-risk patients with uncertain diagnoses who are clinically stable and able to tolerate imaging, cranial MRI could be performed to exclude structural brain lesions and evaluate cerebral edema or white matter microstructural injury. Integrating clinical data, EEG findings, and MRI features into a unified prediction model may improve the early identification of AKI-associated brain dysfunction.

However, the clinical application of multimodal neuroimaging combined with machine learning still faces several challenges. Neuroimaging is difficult to perform routinely in the ICU because of transport risks, sedative effects, long MRI scanning times, and contraindications. In addition, differences among centers, scanners, and operators may introduce data heterogeneity, limiting model generalizability. Although machine learning models based on multimodal MRI can show high diagnostic sensitivity in single-center cohorts, their performance often decreases after external multicenter validation (119). Large public ICU databases, such as MIMIC-IV and eICU-CRD, also lack sufficient neuroimaging data, restricting large-scale multimodal modeling. Finally, the mechanisms underlying the brain–kidney interaction axis remain incompletely understood. Future studies integrating renal function indicators, urine output, IR-related indices, and multimodal brain imaging may help improve risk prediction, guide stratified interventions, and enhance long-term neurological outcomes in patients with AKI.

## 6 Limitations and future directions

Although increasing evidence supports an association between AKI and brain dysfunction, several limitations remain. First, most mechanistic evidence comes from preclinical AKI models, including ischemia–reperfusion injury, sepsis-associated AKI, nephrotoxin-induced AKI, and bilateral nephrectomy models. These studies have clarified important mechanisms such as systemic inflammation, oxidative stress, BBB disruption, microglial activation, uremic toxin accumulation, neurotransmitter imbalance, and IR. However, animal models cannot fully reflect the complexity of human AKI, especially in critically ill patients with comorbidities, infection, hemodynamic instability, sedative exposure, and multiple organ interactions.

Second, current clinical evidence is mainly observational. Although AKI has been associated with delirium, cognitive decline, dementia, depression, stroke, and increased mortality, causal relationships remain unclear. Many confounding factors, including sepsis, hypoxia, hypotension, metabolic disorders, nephrotoxic drugs, renal replacement therapy, and pre-existing kidney or neurological diseases, may also contribute to brain dysfunction. In addition, randomized controlled trials evaluating interventions for AKI-associated brain dysfunction are still lacking, and evidence supporting anti-inflammatory, antioxidant, IR-targeted, neuroprotective, or natural compound-based therapies remains insufficient for routine clinical use.

Third, AKI populations are highly heterogeneous. AKI differs in etiology, severity, duration, reversibility, and need for renal replacement therapy. Different AKI subtypes, such as sepsis-associated, ischemic, cardiac surgery-associated, contrast-induced, and drug-induced AKI, may involve distinct mechanisms. Moreover, neurological outcomes are not uniformly defined across studies, ranging from acute delirium and coma to long-term cognitive impairment, dementia, depression, and reduced quality of life.

Future studies should include large-scale, prospective, multicenter cohorts with standardized AKI definitions, neurological assessments, delirium screening, cognitive testing, and long-term follow-up. Further mechanistic studies are needed to clarify AKI-specific pathways and their interactions with systemic inflammation, oxidative stress, uremic toxins, IR, BBB injury, and microglial activation. Multimodal approaches integrating clinical data, biomarkers, neuroimaging, electroencephalography, and machine learning may improve early prediction and risk stratification, but these models require external validation. Finally, well-designed randomized controlled trials are needed to determine whether targeted therapies can prevent or alleviate neurological complications in patients with AKI.

## 7 Discussion

Research on AKI-associated brain dysfunction remains both promising and challenging. For early identification, two complementary and potentially transformative strategies have emerged: multimodal neuroimaging integrated with machine learning, and IR -related biomarkers. Multimodal neuroimaging techniques, including rs-fMRI, EEG, and optical neuroimaging, enable noninvasive assessment of brain structure, function, and hemodynamics, thereby overcoming some limitations of conventional clinical scales (113, 116, 118). When combined with machine learning algorithms ranging from XGBoost to advanced deep learning architectures, such as the Vision Transformer, as well as explainable artificial intelligence tools, such as SHAP, these approaches can substantially improve the accuracy and clinical interpretability of risk prediction for delirium and other neurological complications (13, 113, 116, 117). Nevertheless, several practical barriers to the clinical application of multimodal neuroimaging combined with machine learning remain to be addressed.

In terms of therapeutic strategies, the TNF α/TNFR1 signaling pathway and MIF has been identified as a key mediator of AKI-associated brain dysfunction. Both genetic deletion and pharmacological blockade these way can significantly ameliorate neuroinflammation and cognitive deficits, providing a clear direction for targeted therapy (28, 45, 87, 90, 93). The marked upregulation of numerous pro-inflammatory cytokines and chemokines during AKI suggests that additional therapeutic targets remain to be identified, which may be further explored through multi-omics approaches and high-throughput screening. Natural bioactive compounds such as EA and SYR exhibit anti-inflammatory, antioxidant, and neuroprotective properties, offering promising avenues for the development of novel therapeutic agents (49, 100). Furthermore, clinical trials targeting AT1R are being explored to evaluate their therapeutic efficacy in cognitive impairment associated with uremic encephalopathy, while the dual effects of low-dose ketamine in alleviating renal injury and depression-like symptoms also warrant further investigation (63, 101).

Overall, with the continued advancement of multimodal imaging technologies, artificial intelligence–based predictive algorithms, and precision-targeted therapies, significant progress is expected in the early identification and intervention of AKI-related brain injury, ultimately contributing to improved long-term outcomes for affected patients.
